# Epstein–Barr virus-associated lymphoma: current understanding and treatment strategies

**DOI:** 10.1007/s44313-026-00127-4

**Published:** 2026-03-07

**Authors:** Hee Young Ju

**Affiliations:** Department of Pediatrics, Samsung Medical Center, 81 Irwon-Ro, Gangnam-Gu, Seoul, 06351 Korea

**Keywords:** Epstein–Barr virus, Lymphoma, Lymphomagenesis

## Abstract

Epstein–Barr virus (EBV) infection is well-known for its high prevalence rate, association with several diseases including cancer and autoimmune conditions, and a wide variety of symptoms and prognosis. When acquired at a young age, primary infections are often asymptomatic; however, in adolescence and young adulthood, symptomatic infections develop, such as in infectious mononucleosis. A special feature of EBV infection is its ability to establish a latent infection in B cells which can lead to long-term infection. Subsequent cellular transformation and viral protein expression can result to EBV-mediated carcinogenesis. Latent proteins expressed by EBV play a role in the pathogenesis of EBV infection and carcinogenesis. These proteins are responsible for a diverse range of functions including cell transformation, cell reprogramming, immune evasion, immune suppression, angiogenesis, cell cycle regulation, and B-cell receptor mimicry.

EBV infection is associated with diffuse large B-cell lymphoma, Hodgkin lymphoma, NK/T cell lymphoma, Burkitt lymphoma, post-transplant lymphoproliferative disorders, and primary CNS lymphoma. The clinical presentation varies depending on the specific disease and EBV status, with EBV-positive lymphomas generally associated with poorer prognosis than EBV-negative cases.

This review aimed to examine the current understanding of the pathogenesis of EBV-associated lymphoma and to evaluate emerging and accepted therapeutic strategies.

## Introduction

The Epstein–Barr virus (EBV) is a large (−175 kb) gamma herpesvirus that infects > 90% of the human population [1]. EBV was initially observed in cultured lymphoblasts from Burkitt lymphoma sample by M.A. Epstein, Y.M. Barr, and B.G. Achong and reported in 1964 [2, 3]. Thus, the association between EBV and lymphoma has been known since its discovery.

EBV infection in early childhood is usually asymptomatic, but can manifest as infectious mononucleosis in adolescents and young adults. Human B cells are the main host cells for EBV, and one of its defining characteristic is its capacity to cause latent B cell infection [4]. EBV latency is thought to be established by a combination of low antigen expression, dedicated immune evasion proteins, and microRNAs [5–7].

EBV-associated cancer can develop when the infection is not well controlled within the B cells or if the virus spreads to other lymphocyte populations, such as T cells and previously mutated epithelial cells. EBV-associated tumors account for approximately 2% of all human malignancies [8, 9].

## EBV and lymphomagenesis

Observations of the geographic distribution of endemic Burkitt lymphoma has improved the understanding of EBV-associated lymphoma pathogenesis. The incidence of endemic Burkitt lymphoma, which is related to co-infection with *Plasmodium falciparum* malaria and EBV, is high in the mid-African damp and humid regions. This finding is explained by the fact that *P. falciparum* malaria promotes prolonged expansion of germinal centers containing activation-induced cytidine deaminase-expressing B cells and a more mature lymphoma phenotype [10]. In addition, *P. falciparum* co-infection might compromise EBV-specific immune control, particularly Epstein–Barr nuclear antigen (EBNA) 1, resulting in elevated EBV viral loads [11, 12]. Other co-infections known to be related to EBV-associated lymphoma include human immunodeficiency virus infection, which suppresses immune responses against EBV, thus promoting lymphoma development [13]. Another unique population is EBV-associated lymphoma, which is related to immune suppression by inborn errors of immunity (e.g., X-linked lymphoproliferative disease) [14]. The host’s genetics and co-infection with disparate pathogens both promote EBV-associated lymphomagenesis by limiting the host’s immune responses.

After transmission via saliva, EBV reaches the mucosal epithelial cells of the host and, after transcytosis, reaches the submucosal lymphoid tissues. Subsequently, EBV infects B cells. Then, the EBV genome circularizes into episomes and starts to express viral anti-apoptotic proteins, viral BCL-2 homologues BamHI-A rightward frame (BARF1) and BHRF1, as well as the latent EBV gene products, EBNA2 and EBNA-leader protein (EBNA-LP). Subsequently, EBNA2 and EBNA-LP amplify the expression of latency-associated EBV gene products (EBNA1, EBNA2, EBNA3A, EBNA3B, EBNA3C, and EBNA-LP), non-translated RNAs, and microRNAs, consequently replicating the viral DNA through host cell proliferation [7]. The produced proteins play important roles in the pathogenesis of EBV infection and carcinogenesis through cell transformation, cell reprogramming, immune suppression, immune evasion, angiogenesis, cell cycle regulation, and B cell receptor (BCR) mimicry [15, 16]. Latent membrane protein (LMP)1 can emulate the signaling of the B cell surface receptor CD40, thereby activating the nuclear factor kappa B (NF‐κB), c-Jun N-terminal Kinase (JNK), mitogen-activated protein kinase (MAPK), Janus kinase/signal transducer and activator of transcription, and phosphoinositide 3-kinase (PI3K) signaling pathways to facilitate B cell proliferation and modulate the immune response for survival. This function is pivotal in EBV infection and latency establishment. During the transition from latent to lytic infection, EBV strategically targets the host Toll-like receptors to diminish the production of type I interferons (IFNs) and the host antiviral activity, thereby evading immune detection. Among the lytic EBV proteins, BamHI Z fragment leftward open reading frame (BZLF1) reduces the responsiveness of intracellular receptors to tumor necrosis factor-alpha and IFNγ) as well as regulates NF‐κB activity and inhibits the expression of IFN-α and IFN-β. By employing various immunomodulatory strategies during its latent and lytic phases, EBV ensures the persistence of its genome and production of viral particles, thereby facilitating infection. Investigating the mechanisms and distinctions of immune regulation during the latent and lytic phases of EBV infection is essential for developing novel therapeutic strategies for managing EBV-associated diseases [17].

**Fig. 1:**
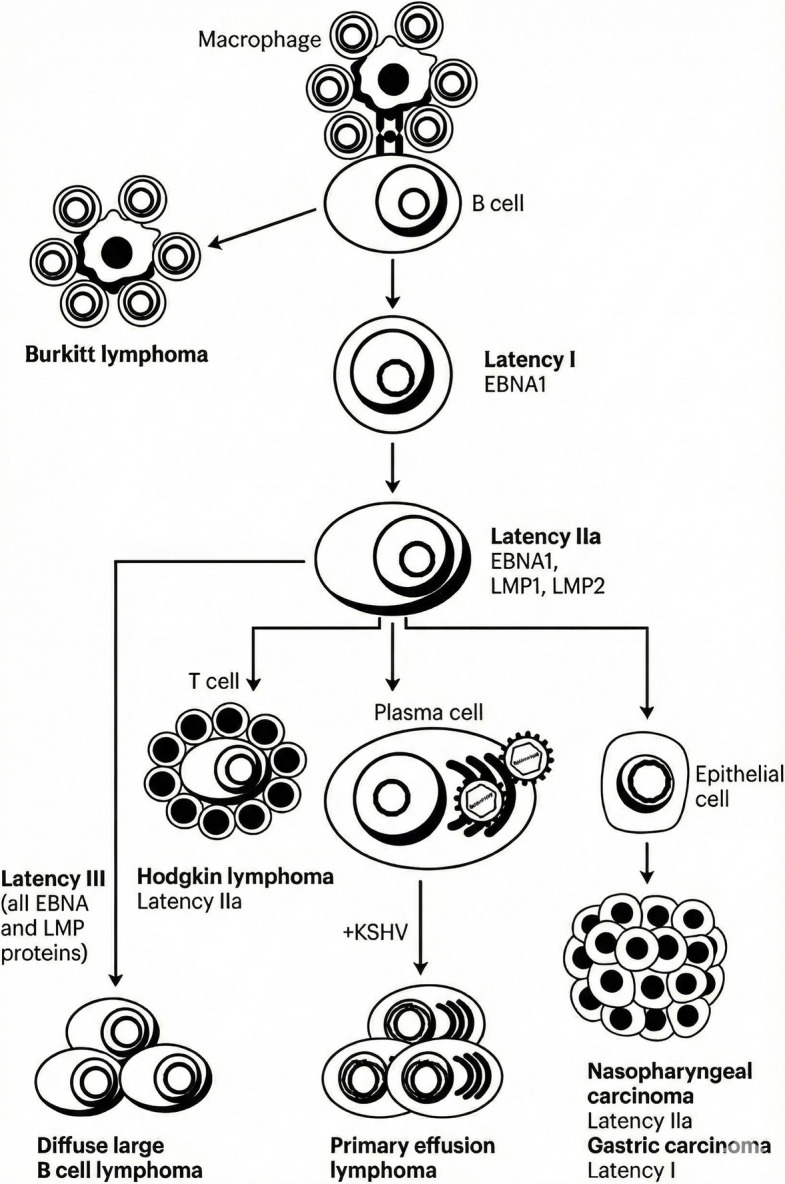
Epstein–Barr virus (EBV)-driven lymphomagenesis. EBNA, Epstein–Barr nuclear antigen; EBNA-LP, Epstein–Barr nuclear antigen leader protein; LMP, latent membrane protein

EBV can establish multiple types of latent infections—latency 0, I, II, and III— with different viral proteins produced at each latency. Malignancies are strongly associated with specific latent states (Fig. 1). Burkitt lymphoma is associated with type I latency, whereas Diffuse Large B-Cell Lymphoma (DLBCL) is associated with type II or III latency. Hodgkin lymphoma and NK/T lymphomas exhibit type II latency, whereas post-transplant lymphoproliferative disorder (PTLD) is usually associated with type III or II latency [18]. Kaposi sarcoma-associated herpes virus-associated primary effusion lymphomas express latency I, while nasopharyngeal carcinoma and gastric carcinoma express latency IIa and I, respectively [7].

Burkitt lymphoma exhibits type I latency, with EBNA1 as the main latency protein. Several studies have shown that EBNA1 miRNAs alter gene expression in multiple cellular processes including apoptosis, transcription, and gene regulation [19]. In addition, EBNA1 knockout results in the apoptosis of EBV-infected cells and leads to episomal loss in Burkitt lymphoma cells, showing that EBNA1 can enhance resistance to apoptosis [20].

DLBCL expresses Type III latency, and EBNA2 has been reported to play a role in oncogenesis [21]. EBNA2 is known to increase Programmed Death Ligand (PD-L)1 expression by downregulating *miR-34a*. In addition, the inactivation of sphingosine-1-phosphate receptor 2 signaling induces constitutive expression of the PI3K pathway [22]. EBV-associated DLBCL expresses increased levels of NF-kB and EBV-mediated STAT3 upregulation, which results to inhibition of B-cell differentiation and downregulation of apoptosis genes [23, 24].

NK/T cell lymphoma expresses type II latency. LMP1 activates NF-kB, which in turn activates peroxisome proliferator-activated receptor-γ (PPARγ) coactivator-1 β (PGC1β), leading to mitochondrial dysfunction and reactive oxygen species generation.

Classical Hodgkin lymphoma is also associated with type II latency. LMP1 reprograms germinal center B cells to resemble the Hodgkin Reed-Sternberg (HRS) phenotype [25]. Moreover, LMP1 activates the PI3K-protein kinase B (AKT)-mammalian target of rapamycin (mTOR) axis to inhibit T cell activation. This leads to enhanced regulatory T cell recruitment and autophagy regulation [26]. The tumor microenvironment (TME) also plays a significant role in classical Hodgkin lymphoma. With LMP2A expression, Syk/PI3K/NF-kB signaling is activated, attracting immune cells into the TME. Furthermore, EBV promotes the migration of regulatory T cells to the TME, providing EBV-infected cells with the ability to downregulate anti-tumor immune responses [27]. Recent studies have shown that although the lymphoma clone loses its EBV genome over time, EBV infection is still detectable using highly sensitive methods, indicating that lymphomagenesis of a subset of EBV-negative lymphomas is related to EBV [28].

## EBV-associated lymphoma: classification

Table 1 summarizes the lymphoma subtypes that incorporate EBV into their diagnostic classification, as outlined in the newly updated World Health Organization (WHO) Classification of Haematolymphoid Tumors (WHO-HAEM) [29]. While EBV may not be explicitly mentioned in the diagnostic classification nomenclature, it is widely recognized that EBV plays a significant role in the pathogenesis of various lymphomas. For example, Burkitt lymphoma has been historically categorized into three subtypes: "endemic," "non-endemic or sporadic," and "immunodeficiency-associated" [30]. However, recent findings indicate that EBV-positive and -negative Burkitt lymphomas represent distinct biological categories based on their molecular characteristics, independent of the epidemiological context and geographic location. EBV-positive and -negative Burkitt lymphomas share coding mutations that affect pathways such as BCR and PI3K signaling, apoptosis, the SWItch/Sucrose Non-Fermentable complex, and G protein-coupled receptor signaling [31–33]. Nevertheless, EBV-positive Burkitt lymphoma demonstrates significantly higher levels of somatic hypermutations, especially in non-coding regions near the transcription start site; contain fewer driver mutations, particularly in the apoptosis pathway; and has a lower incidence of mutations in genes encoding the transcription factor TCF3 or its repressor ID3 than EBV-negative Burkitt lymphoma [31]. Based on these findings, the WHO-HAEM5 advises distinguishing EBV-positive and EBV-negative Burkitt lymphoma. Furthermore, unlike tumors such as Burkitt lymphoma or nasopharyngeal carcinoma, in which the EBV genome is consistently detected, only a portion of patients with classic Hodgkin lymphoma exhibit a persistent presence of EBV. In EBV-positive classic Hodgkin lymphoma, viral latent proteins such as LMP1 and LMP2A deliver essential survival and proliferative signals, whereas in EBV-negative classic Hodgkin lymphoma, similar oncogenic pathways are activated through somatic mutations, particularly involving the NF-κB signaling cascade. These observations indicate that EBV represents one of several alternative mechanisms that rescues BCR-negative germinal center B cells and promotes HRS cell survival [34].

**Table 1:** EBV-associated lymphoproliferative disorders by WHO classifi cation

WHO Classification, 5th edition	WHO Classification, revised 4th edition
***Large B-cell lymphomas***	
EBV-positive diffuse large B-cell lymphoma	EBV-positive diffuse large B-cell lymphoma, NOS
***Lymphoid proliferations and lymphomas associated with immune deficiency and dysregulation***	
EBV-positive mucocutaneous ulcer	(same)
***EBV-positive NK/T-cell lymphomas***	
EBV-positive nodal T- and NK-cell lymphoma	Not previously included
Extranodal NK/T-cell lymphoma	Extranodal NK/T-cell lymphoma, nasal-type
***EBV-positive T- and NK-cell lymphoid proliferations and lymphomas of childhood***	
Severe mosquito bite allergy	(same)
Hydroa vacciniforme lymphoproliferative disorder	Hydroa vacciniforme-like lymphoproliferative disorder
Systemic chronic active EBV disease	Chronic active EBV infection of T- and NK-cell type, systemic form
Systemic form Systemic EBV-positive T-cell lymphoma of childhood	(same)
***Mesenchymal dendritic cell neoplasms***	
EBV-positive inflammatory follicular dendritic cell sarcoma	Inflammatory pseudotumour-like follicular/fibroblastic dendritic cell sarcoma

Excerpt from “The 5th edition of the World Health Organization Classification of Haematolymphoid Tumours: Lymphoid Neoplasms” [29]

## Clinical implications of EBV in lymphoma

EBV-positive lymphomas have fewer driver mutations than EBV-negative cases, especially in genes involved in apoptosis. A recent study comparing the genomic and transcriptomic characteristics of endemic Burkitt lymphoma cases from Uganda and sporadic cases from the United States showed that activation-induced cytidine deaminase (AICDA) activity was much higher in endemic Burkitt lymphoma cases. Increased AICDA activity is reportedly associated with fewer driver mutations [31]. This indicates that EBV-induced immunomodulation plays a role in oncogenesis with a relatively low mutational burden.

Various studies have shown that persistent and high titers of EBV have prognostic significance. In a study of EBV-positive Hodgkin lymphoma, the plasma EBV-DNA viral load correlated with tumor burden, decreasing with chemotherapy but increasing with relapse [35]. It has been reported that plasma EBV-DNA is highly concordant with viral nucleic acid in situ hybridization on tissue sections in Hodgkin lymphoma. Pretreatment plasma EBV positivity was reported to be an independent predictor of treatment failure, and EBV positivity after treatment was related to failure-free survival in patients with Hodgkin lymphoma [36]. The prognosis according to EBV positivity has also been reported for other lymphomas. A multinational study conducted in South Korea, Hong Kong, and Singapore on extranodal natural killer/T cell lymphoma showed that the post-treatment Deauville score and EBV-DNA positivity were independently associated with progression-free survival and overall survival [37].

## Treatment of EBV-associated lymphoma

Different combinations of chemotherapy regimens are used, depending on the lymphoma subtype. Nevertheless, for EBV-positive lymphoma, targeting EBV is expected to improve treatment outcomes. Based on the understanding of EBV lifecycle and oncogenesis, several treatment approaches have been proposed.

During latency, the viral genome persists as episomes in the nucleus and only a limited number of viral genes are expressed. The EBV becomes resistant to nucleoside-type antivirals because the viral enzyme thymidine kinase, which is a target of many antiviral drugs, is not expressed during latency. Consequently, the concept of inducing EBV-infected cell death by promoting the reactivation of EBV from latent to lytic replication, referred to as the lytic induction treatment, has been proposed. An early study on combined treatment with ganciclovir and arginine butyrate, which can induce EBV-thymidine kinase expression during latency, showed an anti-tumor response [38]. EBV reactivation can help control EBV-associated diseases for several reasons. First, EBV reactivation directly induces apoptosis. Upon EBV reactivation, an intrinsic response to cellular injury is triggered, activating NF‐κB and IRF3 signaling, which facilitates apoptosis [39]. Quercetin, a licorice flavonoid, can induce EBV lytic proteins through mechanisms involving the heat shock response and the MAPK/extracellular signal-regulated kinase and MAPK/JNK pathways [40]. MYC is a protein associated with Burkitt lymphoma and is essential for maintaining EBV latency. Disruption of MYC expression by CBL0137 induces EBV reactivation [41].

Epigenetic modification is another method for EBV lytic induction. A preclinical study showed that decitabine induces the expression of viral antigens in a Burkitt lymphoma xenograft model. Treatment of EBV-positive Burkitt lymphoma cells with decitabine, followed by treatment with EBV-specific cytotoxic T lymphocytes, resulted in T cell homing to tumors and inhibition of tumor growth, highlighting the epigenetic factors required to maintain latency in EBV-infected cells [42]. A recent phase 1b/2 study of recurrent EBV-positive lymphoid malignancies treated with nanatinostat (a hydroxamic acid-based class I-selective histone deacetylase inhibitor) and valganciclovir in patients without viable curative treatment options showed a 40% overall response rate, with a median response of 10.4 months [43].

Another approach for treating EBV-associated lymphoma is immune checkpoint inhibition. A preclinical study reported that following EBV infection of naïve B cells, PD-L1 and PD-L2 protein expression are upregulated. PD-L1 blocking with the anti-PD-1 monoclonal antibody, pembrolizumab, resulted in more efficient killing of EBV-infected B cells by EBV-specific cytotoxic T lymphocytes [44]. Furthermore, a positive response was observed when a combination of PD-1 inhibitors and chemotherapy was administered to patients with refractory EBV -associated DLBCL who exhibited resistance to first-line immunochemotherapy regimens [45].

Adoptive T cell therapy, the therapeutic transfer of defined T cell immunity, offers great potential for the treatment of challenging viral infections owing to its high specificity for tumor cells and low off-target toxicity. Both autologous and third-party EBV-specific cytotoxic T-lymphocytes have been considered for difficult-to-treat EBV infections.

A multinational phase II study involving an autologous EBV-specific T cell product (baltaleucel T) for advanced, relapsed extranodal NK/T cell lymphoma showed anticancer activity with an overall response rate of 50% [46]. A more recent global phase 3 trial of off-the-shelf EBV-specific cell therapy for relapsed/refractory EBV-positive PTLD reported excellent survival outcomes, with 50% of the patients achieving an objective response and 100% overall survival at one year [47].

Another approach involves the use of an EBV vaccine; potential targets include gp350, gp42, gH-gL, EBNA1, LMP1, and LMP2. Among the various targets, a monoclonal antibody targeting EBV gp42 has been reported to be effective in preventing lymphomas [48].

## Conclusion

EBV plays a significant role in the pathogenesis of lymphoma, and its mechanisms vary according to the specific subtype. Nevertheless, EBV serves as a valuable predictor of disease activity and prognosis in various lymphoma types. Notably, EBV is characterized by its latent capacity, which suggests that lytic induction can enhance treatment efficacy. In challenging cases of EBV-associated lymphoma, the development of EBV-specific cytotoxic T lymphocyte therapy and vaccines is essential.

## Data Availability

No datasets were generated or analysed during the current study.
